# Design and evaluation of a global workspace agent embodied in a realistic multimodal environment

**DOI:** 10.3389/fncom.2024.1352685

**Published:** 2024-06-14

**Authors:** Rousslan Fernand Julien Dossa, Kai Arulkumaran, Arthur Juliani, Shuntaro Sasai, Ryota Kanai

**Affiliations:** ^1^Araya Inc., Tokyo, Japan; ^2^Microsoft Research, New York, NY, United States

**Keywords:** global workspace theory, attention, embodiment, artificial neural networks, imitation learning

## Abstract

As the apparent intelligence of artificial neural networks (ANNs) advances, they are increasingly likened to the functional networks and information processing capabilities of the human brain. Such comparisons have typically focused on particular modalities, such as vision or language. The next frontier is to use the latest advances in ANNs to design and investigate scalable models of higher-level cognitive processes, such as conscious information access, which have historically lacked concrete and specific hypotheses for scientific evaluation. In this work, we propose and then empirically assess an embodied agent with a structure based on global workspace theory (GWT) as specified in the recently proposed “indicator properties” of consciousness. In contrast to prior works on GWT which utilized single modalities, our agent is trained to navigate 3D environments based on realistic audiovisual inputs. We find that the global workspace architecture performs better and more robustly at smaller working memory sizes, as compared to a standard recurrent architecture. Beyond performance, we perform a series of analyses on the learned representations of our architecture and share findings that point to task complexity and regularization being essential for feature learning and the development of meaningful attentional patterns within the workspace.

## 1 Introduction

While neuroscience had a profound influence on the fields of artificial neural networks (ANNs) and deep learning (DL) in the past (Rosenblatt, 1962; Fukushima, 1980; Rumelhart et al., 1986; LeCun and Bengio, 1995), in recent years the direction of influence has largely changed, and deep neural networks (DNNs) have emerged as a popular model of processing within the biological brain. The success of convolutional neural networks (CNNs; LeCun and Bengio, 1995; Krizhevsky et al., 2012; Simonyan and Zisserman, 2014) in the ImageNet visual object recognition competition (Russakovsky et al., 2015) spurred comparisons between trained CNNs and areas of the brain related to visual processing (Afraz et al., 2014; Seijdel et al., 2017; Pogoncheff et al., 2023). This trend soon extended to investigating similarities between ANNs and the auditory cortex (Pichevar and Rouat, 2007; Szabó et al., 2016; Drakopoulos et al., 2021). Furthermore, the development of ANNs for natural language processing tasks has advanced our understanding of language processing in the human brain. For example, these models have been used as tools to explore and generate hypotheses on the neural mechanisms involved in language comprehension and production (Caucheteux and King, 2022). Similarly, biological research on other aspects of cognition, such as memorization, has benefited from comparative studies between populations of neurons and their artificial analogs (Bedia et al., 2007; Li and Fan, 2019), such as recurrent neural networks (RNNs), which are a prominent class of architecture used to process temporal data (Hochreiter and Schmidhuber, 1997; Cho et al., 2014; Sak et al., 2014). Another type of ANN, the Transformer (Vaswani et al., 2017) has similarly served as an empirically grounded tool to investigate the mechanism of attention and abstraction in the brain (Belinkov and Glass, 2019; Wilterson and Graziano, 2021).

Given the progress in using DL for studying natural intelligence, we believe that now is a prime time to use the latest advances in DL to investigate higher-order thought processes and functions in the brain, such as the access and processing of conscious information (Bengio, 2017; Goyal and Bengio, 2022; Juliani et al., 2022a). In the same way that DL, which can directly process raw inputs such as images or audio, is used to investigate representations in the brain, we are now able to scale up computational models of consciousness. In particular, we focus our efforts on global workspace theory (GWT; Baars, 1993), which is one of the most popular theories of conscious function. This theory was heavily inspired by the structure of the biological brain: the existence of multiple specialized functional networks, and the fact that they process the information flow from the environment in parallel (Baars, 1993, 2005). Early computational implementations of GWT were necessarily limited in their sophistication and what domains they could be applied to Baars (1993) and Shanahan (2006). More recent studies (Goyal et al., 2021; Juliani et al., 2022b; Butlin et al., 2023) have focused on DL models which can achieve behavior consistent with cognitive phenomena related to consciousness, attentional control, and working memory, considered critical components of GWT (Baars, 2005; Goyal et al., 2021; Butlin et al., 2023); however, each of these previous studies have lacked evaluation of the model while embodied within a multimodal environment. The maturation of both leading theories of consciousness and artificial models of cognition therefore warrants additional integration attempts, with the potential for increasing our understanding of both biological and artificial intelligence.

In this study, we followed the recently outlined “indicator properties” of consciousness: criteria for artificial agents to manifest behavior consistent with contemporary theories of conscious function (Butlin et al., 2023). While Butlin et al. (2023) *proposed* indicator properties for various theories of consciousness, these lacked concrete implementation details. Hence, one of our main contributions is designing an agent architecture that satisfies all four GWT indicator properties which they outlined—a feat that they claimed was not achieved by prior artificial intelligence implementations. We then trained this agent to perform audio-guided navigation in a visually realistic 3D environment (Chen et al., 2020, 2021, 2022), which, to the best of our knowledge, is the most realistic setting a global workspace agent has been tested in. We analyzed how the agent's representations compare to a standard DL baseline (Alain and Bengio, 2016; Dai et al., 2022; Zhang et al., 2022), as well as its attentional patterns. Another one of our main contributions is performing an extensive set of experiments over a large range of global workspace sizes, elucidating the impacts of imposing a significant bottleneck on the global workspace size in the development of dynamic patterns of attention.

Our study reveals key insights from deploying a global workspace embodied agent in realistic multimodal tasks. In our chosen task, the global workspace agent performs better and more robustly than the baseline for smaller working memory sizes, although the difference disappears as the size of the bottleneck increases. Beyond this, we believe that a more sophisticated task or environment may be needed to reveal potential behavioral benefits conferred by a global workspace. In particular, this is confirmed by an ablation on the size of the global workspace, as larger agents do not perform significantly better. We also show that the smaller agents, with more of a bottleneck, develop more mixed attention patterns, integrating information from different modalities over time, and all agents primarily use cross-attention across input modalities to perform the navigation task. Finally, an analysis of the weight matrices within the global workspace agent's sensory encoders indicates that these agents prefer to utilize the global broadcast to process information over time, as opposed to the more direct recurrent feedback within the global workspace itself. These revelations highlight the nuanced, consciousness-related processes of artificial agents and emphasize how the global workspace model's attention mechanisms are intricately linked to the size of its workspace. This research paves the way for deeper understanding and development of artificial agents capable of more human-like processing in diverse, sensory-rich environments.

## 2 Materials and methods

### 2.1 Functional theories of consciousness

A prominent division of research topics within the domain of consciousness science is between the study of the so-called “hard problem,” which seeks explanations for why the phenomena of consciousness exist given our physical universe, and the “easy problems,” which consist of explanations for why specific patterns of brain activity correlate with specific states of consciousness (Chalmers, 1995). The related “hard question” provides a third possibility of inquiry, seeking explanations for the functional role of consciousness as it manifests in evolved organisms (Dennett, 2018). This functional approach enables the extension of the study of consciousness from the exclusively physical domain to the virtual domain, where artificial systems with various functional properties can be compared to systems in the physical world which we believe instantiate and in some sense “utilize” consciousness in order to accomplish goals. It is within this domain that theorists have proposed models such as GWT (Baars, 1993, 2005), information generation theory (Kanai et al., 2019), and attention schema theory (AST; Graziano, 2017; Wilterson et al., 2020), among others (Rosenthal, 1993; Juliani et al., 2022a; Butlin et al., 2023).

GWT (Baars, 1993, 2005) is a framework proposed to formalize access consciousness—the idea that what is conscious is information that is accessible across various mental processes (Block, 1995). Given its abstract and functional nature, it has also been theoretically extended to artificial agents as well (Dehaene et al., 2021). GWT firstly posits that the brain consists of numerous specialized information processing modules interconnected with each other, where, as an approximation, these modules can be thought to correspond to functional networks within the brain. The global workspace can then be understood as a common representational space of fixed capacity where the aforementioned modules can share information. It therefore functions as a pivotal bottleneck, only letting through the most salient information originating from diverse input modalities and sources, while integrating them into a coherent representation. The process of information gating itself can be understood as a specific instantiation of internal attentional modulation. In addition, the global workspace can be interpreted as working memory within the brain, as it is also expected to maintain the information required to sustain a state of consciousness across variable lengths of time (Lau and Rosenthal, 2011; Park and Tallon-Baudry, 2014).

Several approaches have been proposed that take inspiration from modern cognitive science and DL to attempt to provide a concrete implementation of a global workspace in an artificial system (Goyal et al., 2021; Juliani et al., 2022b). Juliani et al. (2022b) demonstrated that the Perceiver architecture (Jaegle et al., 2021b) meets the criteria of a functional global workspace as described by Baars (1993). Namely, the proposed Perceiver-based agent structure was empirically shown to satisfy requirements of GWT such as *broadcasting across modules*, *selective attention* and *working memory* over a set of behavioral tasks inspired by those used in the cognitive science literature. However, prior work has been restricted to unimodal input data, namely either visual or textual information (Goyal et al., 2021; Juliani et al., 2022b). Humans, on the other hand, manifest consciousness while navigating the relatively more complex physical world, which is perceived through multiple sensory inputs or modalities. This increased complexity creates strong incentives for the emergence of specialized independent modules, as well as central mechanisms for sharing the relevant information from multiple modules to construct behavior. This multimodality has previously been highlighted as an important aspect of the global workspace (VanRullen and Kanai, 2021). The tenets described above are critical components of GWT (Baars, 1993, 2005; Butlin et al., 2023). Consequently, the investigation of the existence of consciousness in artificial agents could benefit from being conducted in a similar setting.

Despite the breadth of its explanatory power, GWT still leaves many implementation details underspecified, which has resulted in many interpretations by different researchers over time (Baars, 1993, 2005; Shanahan, 2006; Goyal et al., 2021; Juliani et al., 2022b). In their recent position paper on consciousness in AI agents, Butlin et al. (2023) presented a comprehensive overview of various theories of consciousness from a functionalist perspective, including recurrent processing theory (RPT; Lamme, 2006, 2010), GWT (Baars, 1993, 2005), and AST (Graziano, 2017; Wilterson et al., 2020), while also touching upon the aspects of agency and embodiment of such agents. Based on this broad survey of existing theories of consciousness, they produced a list of indicator properties that would be highly correlated with the existence of AI consciousness, from which we compiled the properties relevant to the scope of this study in Table 1.

**Table 1:** Indicator properties relevant to a global workspace agent.

**Property**	**Description**
RPT-1	Input modules are independent and use algorithmic recurrence
RPT-2	Input modules generate organized and integrated perceptual representations
GWT-1	Multiple specialized systems capable of operating in parallel, and independently from each other
GWT-2	Limited capacity workspace, entailing a bottleneck in information flow and a selective attention mechanism
GWT-3	Global broadcast makes information in the workspace available to all modules
GWT-4	State-dependent attention, giving rise to the capacity to use the workspace to query modules in succession to perform complex tasks

While such properties broadly emerge from existing works investigating consciousness in AI and proposing various implementations (Goyal et al., 2021; Juliani et al., 2022b), there is no clear consensus on which method should be used to implement them. In this work, we analyzed the proposed indicator properties for GWT and developed a concrete implementation that explicitly satisfies all of the outlined indicator properties. We note that while there are other possible neural architectures which may also satisfy these properties, our goal here is not to be exhaustive of the space of possibilities, but rather to demonstrate the characteristics of an empirically validated architecture which is consistent with the indicator properties as described above.

### 2.2 Implementations of global workspace theory

While the primary goal of DL methods may not be the creation of conscious agents, some of the algorithms and architectures developed to date share parallels with the high-level cognitive mechanisms exhibited by humans. Because of these parallels, we can find some aspects in which existing DL algorithms and ANN architectures that already align with some of the indicator properties of consciousness, which makes them promising candidates for implementing high-level cognitive mechanisms.

For example, Transformers (Vaswani et al., 2017) are DL architectures heavily reliant on an attentional mechanism, which is a core component of theories of consciousness such as GWT (Baars, 1993; Juliani et al., 2022a) and AST (Graziano, 2017; Wilterson et al., 2020). While Transformer-based architectures have meteorically risen in popularity and become widely used across numerous academic and industrial applications, they lack the overall structure of a system with a global workspace. Transformers use self-attention to integrate information from different positions in a sequence, and from different modules, thus resembling a limited-capacity workspace. However, neither those pre-processing modules, nor the Transformers themselves, are recurrent, and their residual stream not only lacks a distinct workspace integrating other elements as a global workspace would, nor does it make such a workspace accessible to downstream modules (Butlin et al., 2023). The Transformer architecture has been modified to include the addition of a global-workspace-like module (Goyal et al., 2021). In the system of Goyal et al. (2021) there is a sharing of information between multiple modules through a common bottlenecked representation. However, the global workspace presented in this system is not recurrent, meaning that it is unable to satisfy GWT-4.

The Perceiver (Jaegle et al., 2021b) and PerceiverIO (Jaegle et al., 2021a) architectures are consecutive iterations of the seminal concepts of attention-based processing introduced in Transformers, and are better able to satisfy the indicator properties. Namely, the Perceiver and PerceiverIO focus on a limited-capacity latent space to integrate information from specialists, addressing the computational expense of pairwise interactions in self-attention. PerceiverIO employs self-attention and cross-attention to process information in the latent space, allowing it to handle inputs from multiple modalities. An argument could be made that Perceiver-based architectures satisfy the property of having specialized modules (GWT-1), but they only do so implicitly. While they do feature a latent workspace, the Perceiver architecture notably lacks global broadcasting to the independent, pre-processing modules, thus falling short of satisfying GWT-2. With this in mind, Juliani et al. (2022b) proposed a refined implementation of the Perceiver which is more in line with the GWT indicator properties. However, this architecture was applied to unimodal behavioral tasks inspired by cognitive psychology, which can be a limiting factor for the emergence of high-level cognitive processes.

From a distanced perspective, architectures such as the conscious Turing machine (CTM; Blum and Blum, 2022) also offer a model of consciousness, inspired by both GWT and theoretical computer science. While the CTM implementation is concrete and well-defined, it diverges from the GWT model specification. Notable differences lie in either the elimination or simplification of certain GWT aspects, such as implementing direct connections from input to output modules, instead of transiting through a shared workspace. The CTM emphasizes a computational model with predictive dynamics and a multimodal inner language which contributes to its concept of consciousness. From a broader perspective, this model also considers the roles of special processors and the interplay of prediction, feedback, and learning in forming the consciousness experience. We also note that the CTM is purely theoretical and has not been empirically evaluated.

In this study, we design a global workspace agent that explicitly accounts for the GWT indicator properties, thereby including those for RPT. Moreover, we investigate the potential benefits of such architecture in a realistic audiovisual embodied navigation task.

### 2.3 Embodied agents

Embodied agents are agents that have a physical body (real or virtual) with which they interact with their environment (Franklin, 1997). Butlin et al. (2023) emphasized the importance of embodiment for either biological or artificial agents to be endowed with higher-level cognition mechanisms such as memorization, language, planning, reasoning, emotions, consciousness, and manifest their functional properties: namely, the existence of multiple sensory inputs ranging over various modalities, which must be selectively processed to make decisions given a limited computational budget. First, this creates pressure on agents to develop internal (implicit) mechanisms of information processing and integration. Moreover, it requires the agents to leverage internal representations toward the completion of goal-oriented behavior, which is theorized to result in meta-phenomenon such as consciousness (Gibbs, 2005; Baker et al., 2019; Mugan and MacIver, 2019; Blum and Blum, 2022).

Although only tangentially motivated by the goal of recreating consciousness-like phenomena in an artificial context, the field of deep reinforcement learning (DRL) has spurred a plethora of efforts in the development of environments (Baker et al., 2019; Chen et al., 2020, 2021, 2022; Cobbe et al., 2020; Suarez et al., 2021) and agent architectures (Mnih et al., 2013; Schulman et al., 2017; Espeholt et al., 2018; Hafner et al., 2020) that learn to achieve a given goal while navigating through them.

**Figure 1:**
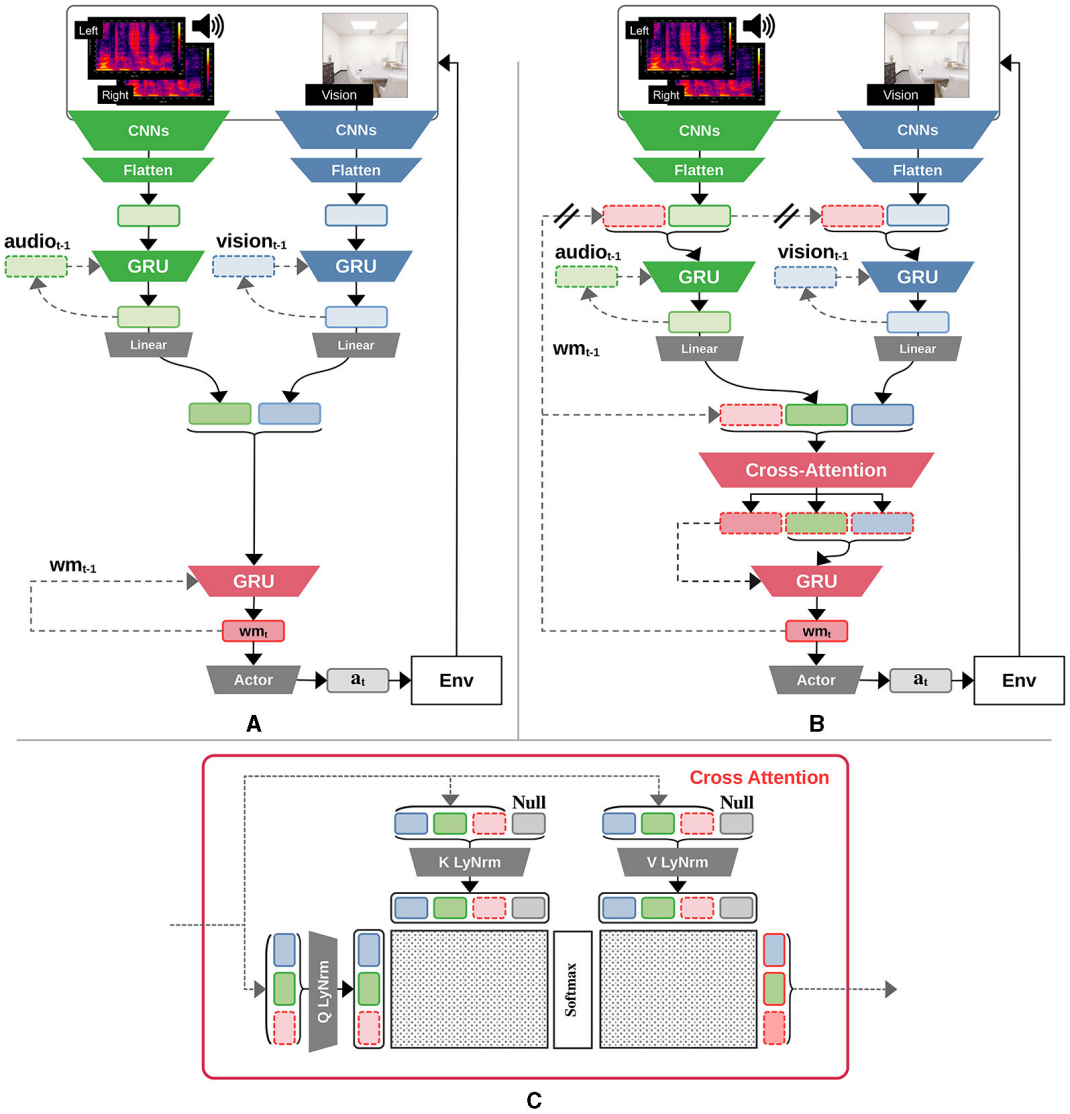
Diagram of agent architectures. **(A)** Baseline GRU agent with recurrent encoders. **(B)** Global-workspace agent satisfying all RPT and GWT indicator properties. The double bars marking the path from the past step's working memory in the recurrent encoder for each modality emphasize the gradient-stopping operation during training that was required to stabilize the learning of this agent variant. **(C)** The proposed cross-attention mechanism takes as input the past step's working memory, the current visual and acoustic features to be used as query sources, and then computes an attention mask for each of those items, along with a null input; this therefore simultaneously incorporates bottom-up, cross, self, and top-down attention. Intuitively, the attention mask determines which information to incorporate from each input.

The core components of such agents include sensory modules which are capable of processing information from different modalities. For example, CNN-based blocks have been leveraged to build agents that can play video games directly from pixels (Mnih et al., 2013; Hafner et al., 2020) or other types of high-dimensional inputs (OpenAI, 2018; Akkaya et al., 2019; Chen et al., 2020, 2021, 2022). Solving some complex tasks also requires the ability to store and process information in a working memory—a capacity which can be made possible through the use of RNNs (Hochreiter and Schmidhuber, 1997; Cho et al., 2014; Hafner et al., 2020). Together, embodied agent components should include (multiple) sensory modules, a working memory, and a policy to take actions in the environment. Sensory modules can be categorized under the umbrella of feature-extracting components, allowing agents to implicitly build representations of their own state, as well as that of the environment. Those representations are then used by downstream components, namely the policy network, which outputs actions that affect the environment (Sutton and Barto, 2018). The policy network is usually implemented as a multi-layer perceptron (MLP), with blocks built out of linear layers and nonlinear activation functions (Goodfellow et al., 2016). Figure 1A illustrates the main components of an embodied agent architecture.

### 2.4 Global workspace agent

In line with GWT-1, we assume that our agent is embodied and experiences a multimodal stream of observations, with each modality being handled by specialized modules that operate in parallel. Given the audiovisual navigation task utilized in this study (Section 2.6), a candidate agent architecture is expected to have an input processing module for each of the visual and acoustic modalities. As motivated in Section 2.3, we leverage CNNs as a basis for encoding information from either of these modalities, which happens to also align with the requirement of having organized and integrated perceptual representations, as stipulated by RPT-2. Each input processing module is then equipped with a gated recurrent unit cell (GRU; Cho et al., 2014) allowing it to leverage a summary of the previously observed information from the same modality (its past state), thus satisfying the condition of algorithmic recurrence as stipulated by RPT-1. The recurrent encoders are augmented with the ability to incorporate the agent's previous working memory, *wm*_*t*−1_, which is processed along with its past state, as illustrated in the upper half of Figure 1B. The working memory is a central, recurrent module that exists in all agents we use in this work, and in the case of our global workspace agent, we consider the working memory to be the current state of the global workspace. This feedback connection therefore satisfies GWT-3, i.e., the concept of a global broadcast that makes the information contained in the workspace available to other modules.

GWT-2 stipulates a limited capacity workspace, entailing a bottleneck in information flow from the input modules into the shared workspace, which is overseen by a selective attention mechanism (Baars, 2005; Juliani et al., 2022b; Butlin et al., 2023). Consequently, our proposed agent architecture is augmented with an attention mechanism (Vaswani et al., 2017), making the overall architecture compliant with both GWT-2, but also introducing a top-down, state-dependent attention from which the workspace can directly query modules to perform downstream tasks. The attention mechanism allows the querying of information that will be passed from both input modalities' features into the working memory for downstream use—for example, to the policy. Making the query mechanism depend on the previous step's working memory, on top of the other two modality components, enables our proposed architecture to satisfy the state-dependent attention criterion, thereby fulfilling GWT-4. To allow a fairer ablation study over the impact of the global workspace as an informational bottleneck, a linear projection is used to match the dimension of the input modality features and the working memory vectors.

Up to this point, due to the nature of the softmax operation in the attention mechanism, the proposed architecture forces the agent to allocate all of its attention over the three inputs (i.e., visual and acoustic features, as well as the working memory). To allow the working memory to be “inattentive”, a *null* input component was added to the key and value components of the attention mechanism, as proposed in RIMs (Goyal et al., 2019). In case there is no salient information that warrants attending to the visual, acoustic, or working memory inputs, the model can (potentially) learn to utilize the null input instead. The detailed attention mechanism is illustrated in Figure 1C.

The proposed mechanism therefore outputs modulated features corresponding to the previous working memory, the acoustic, and the visual modalities, respectively. A central GRU cell will then receive the modulated previous working memory as its hidden state while receiving the modulated acoustic and visual features as inputs to produce the next working memory representation for the current time step, *wm*_*t*_. Analogous to the GRU baseline variant (Section 2.5), the working memory is then passed to the policy (“actor”), as illustrated in the lower half of Figure 1B.

### 2.5 Baseline agent

Our baseline agent is as similar as possible to the global workspace agent while ablating the GWT indicator properties. The simplest of these is to prevent the working memory of the previous step, *wm*_*t*−1_, from being fed to the GRU cell of each input modality encoder, thereby negating GWT-3 (global broadcast); this does however retain RPT-1 and RPT-2.

Altering the overall structure of the agent by having a joint input processing module over both the visual and acoustic modalities would introduce a significant gap when compared to the global workspace agent and the original reference implementation provided in SoundSpaces 1.0 (Chen et al., 2020). Therefore, the independent nature of each input modality encoder is maintained, meaning that the baseline agent does satisfy the GWT-1 property, as illustrated in the upper half of Figure 1A.

All other GWT indicator properties are then negated by removing our proposed cross-attention mechanism, leaving only the central GRU cell. While the total size of the input modalities' features is greater than the size of the state feature vector, hence resulting in a bottleneck in information flow, there is no attention mechanism as required by GWT-2 or GWT-4. Instead, the baseline agent generates the working memory using the unaltered GRU cell. Finally, the working memory is fed to the actor component that produces actions used for either training or evaluation, as illustrated in the lower half of Figure 1A. This baseline agent is thus equivalent to the SoundSpaces 1.0 agent, augmented with recurrent input encoders for each modality.

### 2.6 Multimodal 3D navigation

**Figure 2:**
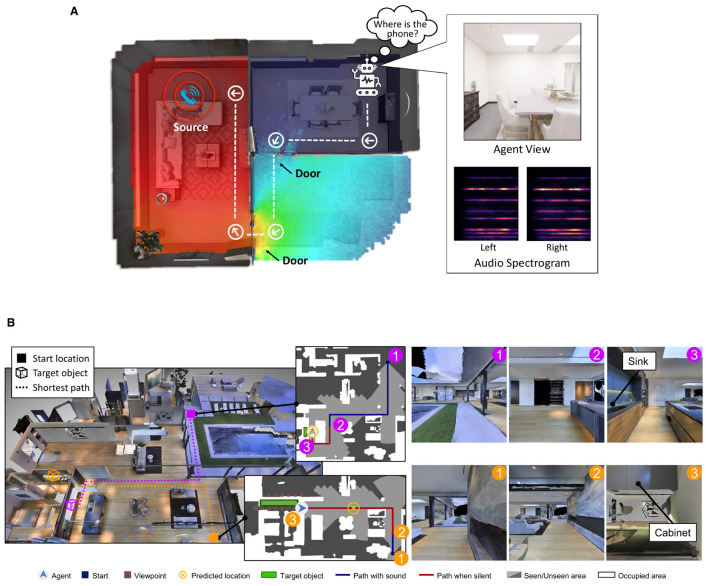
**(A)** Audiovisual navigation task provided by SoundSpaces. The agent has to navigate the rooms to reach the source of the noise (a ringing phone), using a single RGB camera view and binaural audio spectrograms as input. Figure reproduced from Chen et al. (2020) with the authors' permission. **(B)** Two example trajectories of the SAVi (SoundSpaces extension) navigation task. In SAVi tasks, the source of noise can originate from target objects of 21 different categories. In the first trajectory (magenta), the agent hears the sound of a dripping sink, while in the second trajectory (orange), it hears the sound of a cabinet door that is either opened or closed. The duration of the acoustic cues varies. Figure reproduced from Chen et al. (2021) with the authors' permission.

In this work, we approach the study of the global workspace in a realistic embodied task by grounding our experiments in the environments provided by the SoundSpaces library (Chen et al., 2020, 2022) for audiovisual navigation. SoundSpaces introduces a high-quality simulator based on 3D-scanned real-world environments, paired with a novel sound simulation engine able to simulate sound sources and wave propagation in the reconstructed 3D environments. Agents in a SoundSpaces environment are tasked with reaching an object spawned at an arbitrary location following acoustic cues produced by said object, while also using visual information to navigate the 3D environments. The native audiovisual navigation task proposed in SoundSpaces 1.0 (Chen et al., 2020) and 2.0 (Chen et al., 2022) requires the agent to reach the location of a continuously ringing phone, as illustrated in Figure 2A. However, the lack of additional classes of objects that can serve as targets can result in relatively trivial learned representations, while also limiting the emergence of associations between the acoustic and visual modalities. Therefore, we leveraged the semantic audiovisual navigation (SAVi; Chen et al., 2021) extension to SoundSpaces, which incorporates a richer variety of target object categories and their acoustic properties (a dripping sink, moving chair or table, crackling fireplace, and others). Furthermore, the acoustic cues in SAVi are only provided for a variable length duration from the beginning of the episode. Having access to a long-lasting acoustic cue during navigation helps the agent accurately estimate the location of the target location and find the sounding object, as illustrated in Figure 2B (magenta trajectory). However, acoustic cues of a shorter duration (orange trajectory in Figure 2B) only help the agent get a general directional hint, which would force it to commit early acoustic cues into memory and combine them with visual cues to identify the target location. Thereby, SAVi provides a challenging environment with which to test working memory, thus creating a relevant scenario for investigating the properties of an agent with global-workspace-inspired mechanisms.

### 2.7 Agent training

Although the SoundSpaces and SAVi suite of tasks were originally intended for the training and evaluation of goal-oriented RL agents, we used imitation learning, as there are several complexities introduced by RL training on SAVi. Firstly, the reference agent architecture for SAVi relied on additional input fields such as the agent and target's locations—an assumption that can limit extensions to broader settings. The agent also contained manually engineered components specifically geared toward goal-oriented navigation. Despite these additions, the agent was only able to achieve an average success rate of 25%. Moreover, training under the RL paradigm introduces greater variance in the results. Namely, two agent architectures trained under RL might achieve drastically different final performances or learned representations (Lindsay et al., 2021). Compared to other machine learning paradigms, RL agents influence their training data, and will normally observe highly correlated observation-action pairs. Therefore, we adopted the behavioral cloning (BC; Pomerleau, 1988) imitation learning algorithm to reduce confounding factors that might stem from the online RL training paradigm and to ensure a high baseline level of task performance across conditions. BC uses supervised learning on expert trajectory data in order to train an agent. It also guarantees that all agent variants under consideration are exposed to the same observation-action distribution, hereby allowing for isolating the impact of the learned representations on the type of agent architecture, for a fairer and more objective comparison.

To this end, we used the native oracle agent included in the SoundSpaces simulator to generate a dataset D consisting of 500, 000 tuples $\left(o_{t},a_{t}^{*},d_{t}\right)$ of observation, action, and environment termination samples. An observation consists of 128 × 128 × 3 RGB images for the visual modality, and 65 × 25 × 2 spectrograms for the acoustic modality. The action space A is discrete and consists of four actions: allowing the agent to either go forward, turn left, turn right, or stop, which terminates the episode once the target location is reached. All the recurrent components of the agents architectures specified in Sections 2.4 and 2.5 were implemented as GRUs (Cho et al., 2014), with layer normalization (Ba et al., 2016) to further stabilize the learning (Hafner et al., 2020; Yoon, 2023).

For a given agent, with policy π_θ_ parameterized by weights θ, we perform training using minibatches of data sampled from D. Specifically, we sample *B* = 10 (batch size) contiguous trajectories of length *T* = 150 (batch length) from D to produce *N* = *B*×*T* action logits ${\left\{{\hat{x}}_{i}=\pi_{\theta }\left(o_{i}\right)\right\}}_{i=1}^{N}$ via the forward pass. The base BC algorithm optimizes the cross-entropy between the action distribution and the oracle action, $a_{t}^{*}$. We augment this with an entropy regularization term, scaled by a coefficient η = 0.2, to mitigate overfitting of the agent's policy (Kang et al., 2018; Eysenbach and Levine, 2021). Thus, the agents are trained to minimize the following objective function (Equation 1):


(1) $${ℒ}_{\text{BC}}(\theta )=-\frac{1}{N}\sum_{i=1}^{N}\sum_{j=1}^{\left|A\right|}\text{log}\frac{\text{exp}({\hat{x}}_{i,j})}{\sum_{c=1}^{\left|A\right|}\exp({\hat{x}}_{i,c})}\cdot a_{i,j}^{*}+\eta ℋ(\pi_{\theta }),$$

where |A|=4 is the size of the action space, and H(π) denotes the entropy of the policy.

In order to find the best hyperparameter settings for a range of working memory sizes, for both the baseline (GRU) and global workspace (GW) agent, we performed Bayesian hyperparameter optimization using Weights & Biases (Biewald, 2020). For the shared modules, i.e., the audio and visual modality encoders, central GRU cell, and policy network, the same hyperparameters were used across all agents. We tuned the learning rate ∈[0.0001, 0.005], the entropy coefficient η∈[0, 0.5], and the maximum gradient norm (gradient clipping) ∈[0.25, 10], based on ranges commonly observed in the DRL literature (Dhariwal et al., 2017; Raffin et al., 2021; Huang et al., 2022). Each hyperparameter search was conducted over 10 runs, using 25, 000 iterations, after which time the majority of performance could be reached. For the final experiments, we used the optimized hyperparameters (Table 2) for five runs with different seeds, running for 50, 000 iterations. Each run (50, 000 iterations) takes ~5 days on an NVIDIA RTX 3090 GPU, requiring more than 8,616 GPU hours in total for the final experiments.

**Table 2:** Optimized hyperparameters for each agent architecture type and working memory size.

**Model**	**GRU**					**GW**				
**Working memory size**	32	64	128	256	512	32	64	128	256	512
Learning rate (10^−3^)	3.526	2.881	3.148	2.581	0.403	1.231	1.902	0.328	4.502	1.248
Entropy coefficient	0.497	0.066	0.221	0.055	0.363	0.420	0.223	0.396	0.200	0.331
Max gradient norm	2.349	8.999	6.728	7.127	8.387	1.890	2.696	1.704	4.817	7.505

Diverging from the reference work by Chen et al. (2020), the dimension of each modality's feature vector, as well as that of the working memory was set to 64. A critical implementation detail to stabilize the learning of the global workspace agent was to detach the gradients flowing from the GRU cell of each modality encoder into the previous step's working memory *wm*_*t*−1_, as illustrated by the two bars on the corresponding arrows in Figure 1B. All code to support our experiments and analysis can be found at https://github.com/arayabrain/multimodal-global-workspace-agent.

### 2.8 Agent analysis

We employ a multifaceted approach to evaluate and interpret the performance, learned representations, and attention mechanisms (when applicable) of the agent variants under consideration. These three distinct analysis methods enable us to shed some light on the inner workings of the global workspace agent architecture.

#### 2.8.1 Performance evaluation

In DRL, a straightforward approach to validate a given agent architecture is to monitor the episodic return or an equivalent success metric during the agent's training. Consequently, each agent is evaluated every 100 training iterations, using a deterministic policy where the action with the highest probability is always picked. Each evaluation phase consists of collecting the success score (1 if the agent has reached the target location and executed the stop action, 0 otherwise) over five episodes, which are continuously appended to a first-in-first-out list of size 50. To analyze the final performance and sample efficiency of the agent variants, we followed best practices and computed the interquartile mean (IQM; Agarwal et al., 2021), as it is robust to outliers. We calculate the IQM across the latest 50 evaluation episodes, then over the five seeds, and report the final IQM ±95% confidence interval (CI) using 2, 000 bootstrap samples.

#### 2.8.2 Probing learned representations

The SAVi task (Chen et al., 2021) features two semantic concepts that can be queried from the features learned by the agent. This informs us of how well information about the state of the environment is integrated into the learned representations. First, the *target object category*, which varies across episodes, is provided to the agent via the acoustic modality observation, and is available only for a variable duration from the start of the episode. Intuitively, this covers scenarios such as *briefly-ringing doorbell, a door either opening or closing*, and other frequently occurring situations in the real world. This information is crucial to success, as the agent must narrow down the goal to the target object, and the binaural audio also indicates the general location of and distance of the agent from the goal location. There are 21 classes of *target object categories* in total, covering various daily life objects such as chairs, tables, cabinets, sinks, and more (Chen et al., 2021). Second, the *scene* (room) in which an episode takes place can also be obtained from the simulator, as well as inferred from the visual observation. While this information is ancillary to solving the SAVi task itself, it can serve as a proxy to measure how well visual information is integrated into the shared workspace. In total, there are 56 *scenes* in the training dataset.

Probing neural network representations involves evaluating and analyzing internal representations within a pre-trained neural network to understand the learned features and information encoded at different layers. It is thus an invaluable tool for interpreting the inner workings of ANNs and uncovering the latent knowledge encoded in their parameters. Namely, we can use probing to investigate how well information about either the *target object category* or the *scene* is integrated into intermediate layers of the investigated agent architectures, i.e., the learned visual and acoustic features, as well as the working memory. Various works (Pasukonis et al., 2022; Zhang et al., 2022) employ relatively simple MLP architectures conditioned on intermediate network features and trained via supervised learning to classify concepts of interest, then use the classification accuracy as a metric of effective information representation. Intuitively, attaining high classification accuracy using *linear probes* suggests informative and unambiguous learned representations since the concepts of interest can be reliably classified with such simple function approximators (Zhang et al., 2022). Conversely, features that do not encode enough information to predict the target category with a higher than chance accuracy can be deemed uninformative to complete the task at best, and detrimental at worst. We therefore adapt this methodology to train probe networks over features extracted from the fully trained agent architectures under investigation to classify either the *target object category* or the *scene*. Note that the probe training happens independently of the agent's training, thus excluding any form of information leakage from the additional labels programmatically extracted from the environment.

Let *X* = {*vis, aud, wm*} denote the set of candidate input features for the probes corresponding to visual features, acoustic features, and the working memory respectively. Let *Y* = {target object category, scene} denote the set of candidate probe targets. For each pair (*x, y*)∈{*X*×*Y*}, we define a probe network $\xi_{\phi }^{\text{y}}\left(x\right)$ parameterized by weights ϕ, which produces the logits *z*^*y*^(*x*). In practice, each probe is defined as a 2-layer MLP with ReLU activations (Fukushima, 1975). Although a two-layer MLP is not a linear function approximator, recent works (Pasukonis et al., 2022) demonstrated that probes with higher expressive power are beneficial and sometimes even necessary for interpreting complex learned representations. The probe training leverages the same training dataset D that was used in Section 2.7. We first extract *N* of each of the visual features, acoustic features, and working memory representations that correspond to one batch of observations. Each probe then receives a mini-batch of size *K* = 30 of the appropriate input features *x*, and is trained to minimize the cross-entropy loss corresponding to its target *y* over 3, 250 iterations, using the Adam optimizer (Kingma and Ba, 2014) with a learning rate of 2.5 × 10^−4^. Finally, we evaluate each probe's accuracy over a held-out evaluation set of 150 trajectories spanning six different *target object categories* and five *scenes*, and report the classification accuracy.

#### 2.8.3 Attention weights

The exploration of attention weights produced in attention mechanisms proves invaluable in unraveling the intricate interplay between inputs of Transformer-based models (Bahdanau et al., 2014; Clark et al., 2019; Caucheteux and King, 2020). With the capability to concurrently focus on distinct segments of the input modalities, attention becomes a powerful tool for understanding information processing from different modalities, and their integration into the global workspace. Therefore, one key aspect attention weights help us investigate is the dynamic nature of inter-modality interactions. By scrutinizing these weights, we aim to discern whether specific modalities consistently capture the same content from input modalities, unveiling the nuanced relationships between different types of information. Moreover, attention weights can also serve as a quantitative metric for gauging the importance of each modality. Namely, the higher the attention weight, the greater the significance attributed to a particular modality or feature. This quantitative measure would thus allow us to identify crucial elements involved in the decision-making of the proposed global workspace agent.

#### 2.8.4 Contribution of the global workspace broadcast

The broadcast operation is a critical component of GWT. It is designed to share available information across different cognitive processes and modules, allowing them to either prioritize or ignore some local stimuli to the benefit of the overarching objective. As illustrated in Figure 1B, the broadcast is implemented by passing the working memory from the previous time step to the memory cell of the visual and acoustic encoders, respectively. We investigate the general contribution—or lack thereof—of the global workspace at the local level of input processing. To this end, we compute the average magnitude of the linear layer's weights, normalized by the average magnitude of the input features, split on whether they map either the input modality or the past working memory to the encoder recurrent cell. The relative magnitudes give us a basic measure of the relative importance of the bottom-up vs. top-down inputs. By normalizing by the average magnitude of the inputs, we account for the audio or vision features having a different distribution to that of the working memory features.

## 3 Results

### 3.1 Performance evaluation

**Figure 3:**
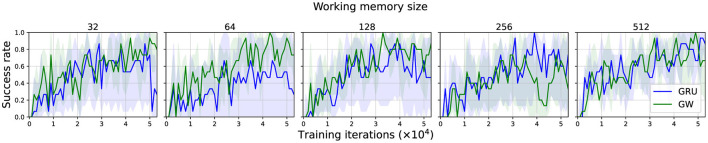
Success rate of each agent type, across a range of working memory sizes. We report the IQM ±95% CI of the rolling average over the last 50 evaluations, aggregated over five independent runs (random seeds). The performance of the global workspace agent is more robust, particularly for smaller working memory sizes.

Figure 3 shows the success rate of each agent type over the training process grouped by their working memory sizes. As detailed previously (Section 2.8.1), we calculated the IQM ±95% CI of the rolling average over the last 50 evaluations. The global workspace agent is relatively robust, achieving ~80% performance across all memory sizes, apart from size 256, where some runs performed poorly; we believe that given the rest of the results, these could be outliers. For reference, a random policy achieves 0% success, while the original SAVi agent trained with RL only reached 25% success on average (Chen et al., 2021). In comparison to the global workspace agent, the baseline GRU agent was less robust, achieving only over 60% performance during the course of training across all memory sizes, and only becoming more stable and competitive with the global workspace agent at higher working memory sizes. Performance sometimes declines after the halfway point, which may be an artifact of our hyperparameter optimization runs stopping at this point to reduce the computational requirements by ~4,000 GPU hours. Regardless, the negligible difference observed across different working memory sizes for the global workspace agent implies that the capacity of the short-term memory required by the task is relatively small. Thus, SAVi is not an adequate benchmark for testing working memory *capacity*.

### 3.2 Probing

**Figure 4:**
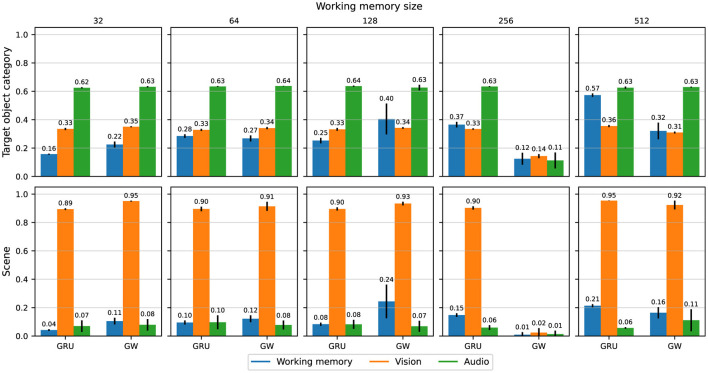
Probing accuracy for the *target object category* and *scene*, conditioned on the working memory, visual features, and acoustic features respectively, grouped by working memory sizes and agent variants. Accuracies are averaged over all evaluation trajectories and five independent runs, with error bars representing ±1 standard deviation.

Figure 4 shows the probing accuracy for the *target object category* (top row) and the *scene* (bottom row), averaged over the held-out evaluation trajectories and grouped by agent architecture.

First, we can ascertain that information relating to the *target object category* is indeed prevalent in the features produced by the acoustic modality encoder (green bars in the top row of Figure 4), given the relatively high accuracy in predicting the category based on said features. The baseline agent does retain more information about the *target object category* in working memory as the size of the memory increases, whereas there is no trend for the global workspace agent. However, there is no other clear difference between the agents, and neither is there any obvious relationship to performance (Section 3.1). Surprisingly, the classification accuracy of the *target object category* given visual features ranges from 14 to 37%, which is significantly above chance accuracy of 1/21 ≈ 4%, thus suggesting that parts of the visual observations also provide *target object category* related information. Indeed, we can expect the agent to be exposed to some information about the *target object category* (e.g., chair) as they come within its visual field of view.

**Figure 5:**
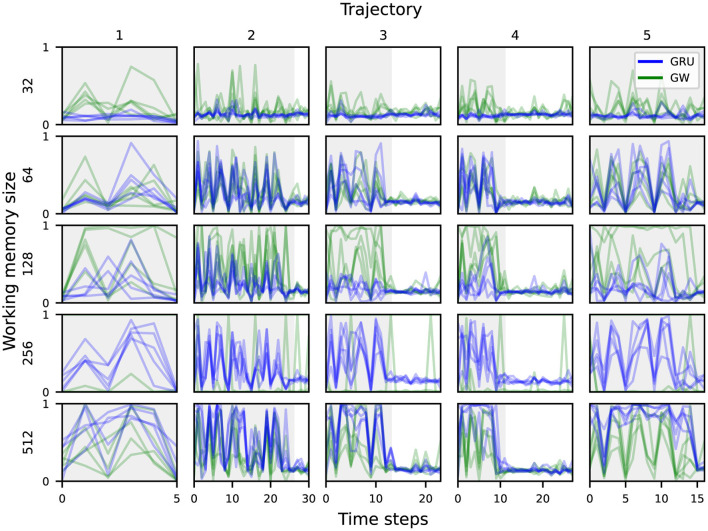
Probing accuracy for the *target object category* based on the agent's working memory, across five different trajectories. The gray area delineates the part of each trajectory where the acoustic cues are playing.

Analogously to the *target object category*, the highest probing accuracy for the *scene* comes from the visual features. This also aligns with our intuitive understanding of an audiovisual navigation task, in that the visual modality is the most important when it comes to identifying which room the agent is navigating through. There is no particular trend in probing accuracy across working memory sizes or between the baseline and global workspace agents. The lowest accuracy is 4% for the smallest baseline agent, which is only slightly above chance (1/56 ≈ 1.79%). As the task can be solved without knowledge of the scene's identification or its layout, this is perhaps to be expected. When it comes to predicting the *scene* based on the audio features, the variants achieved between 6 and 14%. This is still higher than chance accuracy, and might be attributed to specific acoustic cues that uniquely characterize some *scenes*, such as reverberation patterns that depend on the room's layout (Chen et al., 2020, 2021, 2022). While most agent variants managed to integrate enough information from both visual and acoustic modalities into their working memory to succeed at the task, the global workspace agent with memory size 128 seems to achieve the best information integration from both modalities. Figure 5 shows the probing accuracy based on the working memory for the *target object category* over five unrolled trajectories, grouped by working memory sizes and agent types. The gray area delineates the part of each trajectory where acoustic cues are playing. We observe that both agent variants encode the relevant information in their working memory sufficiently enough for reliable prediction when rich acoustic cues are playing. However, once the sound is cut off, the prediction accuracy drops abruptly for both variants. For smaller memory sizes ( ≤ 128), the global workspace agents exhibit higher probing accuracy on average, when compared to their GRU counterparts. This suggests that the global workspace agents are better at integrating information from the input modalities, allowing for consistent performance and robustness across memory sizes (Section 3.1). While this does suggest that neither the conventional GRU nor the proposed attention mechanism help in preserving a high enough probing accuracy for the *target object category* after the sound cutoff, this does not seem to affect the final performance of the agents, as illustrated in Figure 3. In some cases, the agent may already be in a location where the task can be solved purely through visual navigation, and of course, none of the agents achieve a 100% success rate. This nevertheless contrasts with the hypothesized ability of the global workspace to sustain a representation over time.

**Figure 6:**
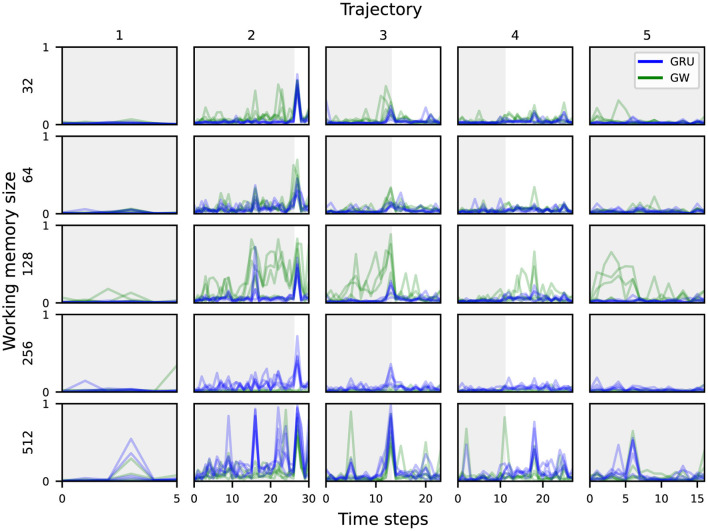
Probing accuracy for an episode's *scene* based on the agent's working memory, across five different trajectories. The gray area delineates the part of each trajectory where the acoustic cues are playing.

Figure 6 shows the probing accuracy based on the working memory for the *scene* over five unrolled trajectories, grouped by working memory sizes and agent types. As was already shown in Figure 4, all of the global workspace agents encode some information from the visual modality into the working memory, which translates into a higher probing accuracy for the *scene* concept over time.

### 3.3 Attention weights

**Figure 7:**
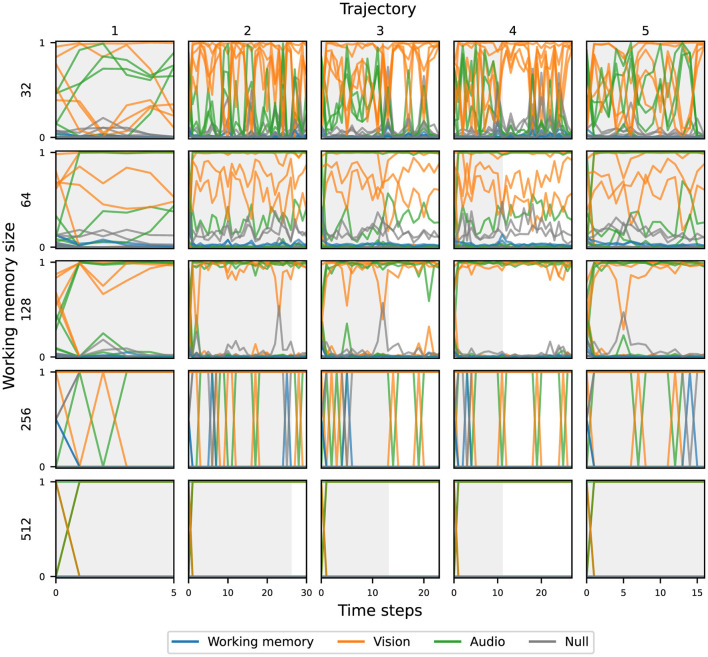
Overview of the attentional patterns of the global workspace agents, based on the working memory query, over five unrolled trajectories.

Figure 7 shows the attention weights resulting from the cross-attention based on the working memory query, across five unrolled trajectories, and five independent runs (seeds), grouped by the working memory size. For the same working memory size, there are no consistent attention patterns across all runs; each agent develops unique strategies to attend to the input components. However, there is a clear pattern as working memory size increases—attention becomes more binary (less mixing of modalities) and the change in attention access decreases—with attention for the largest agents essentially saturating at the beginning of the episode. The attention weights resulting from the cross-attention based on either visual or audio queries follow similar trends. Despite the differences in attentional patterns, all agents were still able to achieve similar task performance. This suggests that with a small bottleneck, agents are forced to use dynamic attention patterns, but given sufficient capacity, there is no pressure to do so.

**Figure 8:**
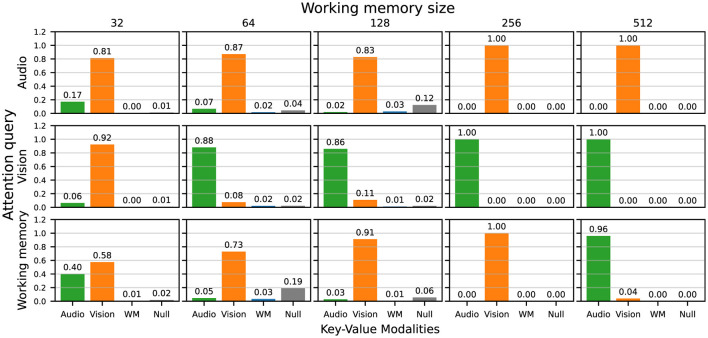
Attention weights averaged over all 150 evaluation trajectories and independent runs (seeds) for each query and key-value combination, grouped by working memory size.

For a more comprehensive view of the attention weights, we investigated the average attention weights over all 150 evaluation trajectories for each query and key-value combinations, grouped by working memory size, shown in Figure 8. In general, the audio queries largely map to the vision keys, and the vision queries largely map to the audio keys, which means that the agents largely perform cross-attention across input modalities. The working memory is matched with either one, or both, of the input modalities. Notably, the working memory and null keys are largely unattended to, potentially implying that most of the important information lies with the current inputs. However, due to the global broadcast, past information can also influence the final stage of sensory input processing.

### 3.4 Contribution of the global workspace broadcast

**Figure 9:**
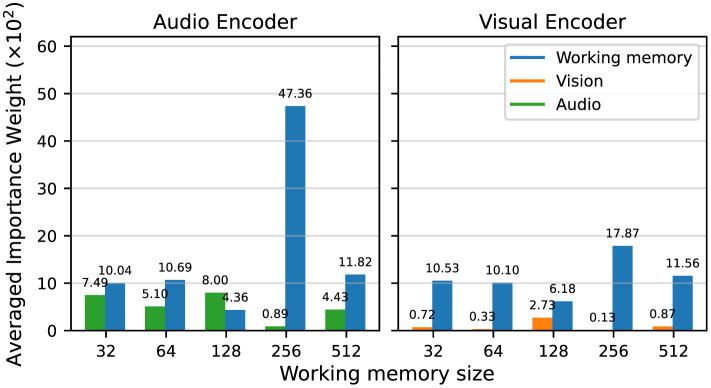
Normalized average magnitude of the input weights for the audio and vision recurrent encoders, for the sensory inputs vs. the previous working memory, grouped by working memory size.

Figure 9 documents the normalized magnitude of the linear layer mapping the input features (audio or vision) and the previous working memory *wm*_*t*−1_ to downstream representations for the recurrent encoders in each sensory module. By this measure, the previous working memory is prioritized over the current sensory inputs, for both the audio and vision encoders. Although at first, Figure 8 appears to show the importance of information from the current time step, these results indicate that the agents are in fact using the global broadcast to propagate information over time in order to solve the audiovisual navigation task.

## 4 Discussion

Motivated by a functionalist approach to consciousness, we introduced a concrete implementation of an embodied agent architecture that fulfills the indicator properties of GWT, as delineated by Butlin et al. (2023). We then proceeded to investigate the benefits, or lack thereof, of such an architecture in a realistic audiovisual navigation task (Chen et al., 2020, 2021, 2022), via feature probing and inspection of learned weights and attentional patterns.

The global workspace architecture does seem to confer improved performance and robustness at smaller working memory sizes. In fact, the performance of the global workspace agents is similar across sizes, and hence we believe future work should investigate more challenging domains. In line with our initial experiments on the SoundSpaces 1.0 task before moving to SAVi, this hints at the possibility that a more complex environment and task definition are required to properly investigate consciousness-related properties and other high-level cognition mechanisms in artificial agents. For instance, Mugan and MacIver (2019) argued that the massive increase in the complexity of terrestrial habitats as compared to aquatic ones is likely to have played a role in the development and emergence of higher-level cognitive abilities such as planning, reasoning, and consciousness in land-based mammals. Similarly, Blum and Blum (2022) emphasized the importance of resource limitations when studying consciousness and related concepts in artificial agents. This is echoed in our ablation study on the capacity of the working memory, which suggests that a stricter informational bottleneck induces more pressure for the selective attention mechanism, incentivizing the latter to learn more dynamic attention strategies over the input modalities. Our study highlights the need for consciousness research to further define the tasks and environment in which candidate architectures for conscious agents and high-level cognitive mechanisms can be suitably evaluated. Unfortunately, the development of realistic simulator environments for training and testing artificial agents is a labor-intensive effort, and beyond the scope of this work.

Two alternative possibilities also exist. The first is that there is a trivial evolutionary advantage conferred by the global workspace. The second is that the “indicator properties” outlined by Butlin et al. (2023) are not sufficiently detailed to capture the unique computational advantages provided by a global workspace in biological organisms such as humans. Given the energy expenditure required to operate the cortex, along with the preservation of workspace-like dynamics over millennia, the latter might be more likely than the former. As such, a fruitful area of future work may be to refine the indicator properties using additional insights from computational and experimental neuroscience.

Potential avenues for future work would thus be to further investigate the role of the global workspace as a bottleneck in the proposed architecture, as well as other quantitative and qualitative properties, such as the connectivity patterns in the inputs and their representations in the shared workspace, or the emergence of cross-modal analogies that encode shared abstract concepts across modalities.

## Data availability statement

The raw data supporting the conclusions of this article will be made available by the authors, without undue reservation.

## Author contributions

RD: Conceptualization, Data curation, Formal analysis, Investigation, Methodology, Software, Visualization, Writing – original draft, Writing – review & editing. KA: Conceptualization, Investigation, Methodology, Project administration, Visualization, Writing – original draft, Writing – review & editing, Formal analysis. AJ: Methodology, Writing – review & editing. SS: Supervision, Writing – review & editing. RK: Funding acquisition, Supervision, Writing – review & editing.
